# Congress of Legal Medicine

**Published:** 1878-10

**Authors:** 


Congress of Legal Medicine.—This was one of the many congresses at Paris this summer, says the "Medical and Surgical Reporter." It passed the following resolutions: "That the Government be asked to establish in France institutions which have been at work for years in Hungary, Austria, Prussia, Belgium, Holland, etc., having for their object the formation of experts in legal medicine and experts in toxicology, remunerated by the State, and who shall be elected by *concours*, having for its basis trials essentially practical; that, with the object of educating such experts, a great extension should be given in the different faculties of medicine to courses of practical legal medicine, such as M. Devergie first established at the Morgue in 1834, and which has been re-opened last year at the requestiof Professor Vulpan, Dean of the Faculty; and that special oourses for instruction in toxicological analysis be opened at the Ecoles de Pharmacie.

